# Recent Insights on Prevalence and Corelations of Hypoactive Delirium

**DOI:** 10.1155/2015/416792

**Published:** 2015-08-10

**Authors:** Vaios Peritogiannis, Maria Bolosi, Charalampos Lixouriotis, Dimitrios V. Rizos

**Affiliations:** ^1^Private Practice Sector, 45444 Ioannina, Greece; ^2^Intensive Care Unit, Hatzikosta General Hospital, 45445 Ioannina, Greece; ^3^General Hospital of Livadia, 32100 Livadia, Greece

## Abstract

Delirium is a complex neuropsychiatric syndrome which is common in all medical settings. It often goes unrecognized due to difficulties in the detection of its hypoactive variant. This review aims to provide an up-to-date account on recent research on hypoactive delirium (HD). Thirty-eight studies, which were conducted in various clinical settings, including the Intensive Care Unit (ICU), were included in this review. Those studies involved recent research that has been published during the last 6 years. Prevalence of HD was found to vary considerably among different settings. HD seems to be more common in critically ill patients and less common in patients examined by consultation-liaison psychiatric services and in mixed patient populations. The presence of HD in ICU patients was associated with higher short- and long-term mortality and other adverse outcomes, but no such association was reported in other settings. Research on other possible associations of HD with clinical variables and on symptom presentation yielded inconclusive results, although there is some evidence for a possible association of HD with benzodiazepine use. There are several methodological issues that need to be addressed by future research. Future studies should examine HD in the primary care setting; treatment interventions should also be the objective of future research.

## 1. Introduction

Delirium is a complex, multifactorial neuropsychiatric syndrome comprising a broad range of cognitive and neurobehavioural symptoms that is common in medical-surgical patients and those in hospice and nursing homes [1]. The clinical importance of delirium is that it is associated with increased morbidity and elevated mortality rates; with longer lengths of hospital stay and nursing home placement; and with poor functional recovery [2, 3]. However, recent evidence in the Intensive Care Unit (ICU) suggests that although delirium prolongs patients' stay in the ICU it does not cause death in critically ill patients [4]. It is unclear whether delirium is a marker of poor prognosis or causally linked to mortality in the ICU [4]. Despite its clinical relevance it is widely accepted that delirium is an underrecognized condition, particularly due to difficulties in diagnosing the hypoactive variant of the syndrome [5–8].

The concept of delirium subtypes has been originally introduced by Lipowski [9] who suggested the use of the terms “hyperactive” and “hypoactive” for the description of patients' motor behavior (restlessness and aggression versus low vigilance and apathy). A third “mixed” category was later added to include patients showing symptoms of both subtypes [10]. Subsequent work has confirmed the clinical utility of delirium subtypes, and two systematic reviews on this topic have been published a decade ago [11, 12]. More recently Meagher [13] presented a detailed account on motor subtypes of delirium and stated that although delirium is a unitary syndrome, delirious patients may differ considerably in phenomenological profile, underlying causation, treatment response, and prognosis. Several studies suggest that delirium occurring in the context of metabolic disorders or organ failure is mostly hypoactive in presentation, whereas delirium due to substance intoxication or withdrawal is more frequently hyperactive [13]. Delirium subtypes may also differ with regard to complications. It has been reported that hypoactive patients are more likely to develop pressure sores or hospital-acquired infections, while falls are most likely in patients with hyperactive delirium [14]. Regarding prognostic significance of delirium subtypes studies have yielded contradictory results [13], but there is some evidence that hypoactive patients may have higher mortality risk compared to other psychomotor activity patient groups [15].

There is an ongoing research on delirium subtypes and here we intend to provide an up-to-date review of the recent literature focused on hypoactive delirium (HD). We focused only on HD because there is evidence that this type of delirium is often missed in everyday clinical practice [16, 17] and misdiagnosis may significantly impact on therapeutic interventions. Conceivably, physicians, nurses, and other health professionals should be aware of and regularly inquire about HD in all treatment settings.

## 2. Sources of Information

A search in the database of PubMed was conducted, for English-language articles published from 2009 to December 2014, with the combination of each of the search terms “delirium,” “hypoactive delirium,” and “delirium subtypes.” We limited our search to recently published work, since the publication of Meagher's comprehensive review (2009) which comprised a total of 34 studies published till 2008 [13]. References cited in the originally retrieved publications were searched to identify additional potentially relevant studies. Only prospective studies in which delirium identification and subtyping were clearly described were considered. No limits were set in the number of participants in the studies to be included in this review. Studies were included regardless of their objective (long-term outcome, patients' quality of life, stability of diagnosis, and a scale's validation) if at least they referred to the prevalence rates of hypoactive delirium.

## 3. Hypoactive Delirium in Different Wards

A total of 38 articles were included in the review involving 4282 delirious patients. Different methods, criteria, and rating scales were used in the study of delirium subtypes. Most researchers have used the Delirium Rating Scale (DRS) [18], DRS-R-98 [19], Confusion Assessment Method (CAM) [20], and CAM-ICU [21] for delirium diagnosis, while for subtyping the most widely used scale was the Ritchmond Agitation-Sedation Scale (RASS) [22]. Results are presented in Tables 1–7. Each table refers to a different study setting and presents briefly the main findings of studies. Percentage rates of HD refer to the total of delirious patients, not to the total of patients.

*Anaesthesia and Cardiac Surgery*. Five studies were conducted in these settings (Table 1). These reports consistently used CAM-ICU and RASS for delirium diagnosis and subtyping, respectively. The reported HD prevalence was high (56–92%). In two studies [26, 27] the development of HD was associated with benzodiazepine use at admission. 

*Consultation-Liaison (C-L) Psychiatry*. There were four recent studies on delirious patients been examined by the C-L psychiatric service, which reported relatively low prevalence of HD (6.25–30%, Table 2). Those studies used different diagnostic tools and subtyping criteria. Differences in symptomatology across delirium subtypes were inconsistently observed in two studies [30, 31]. 

*Hip Fracture Patients*. Four studies presented in Table 3 addressed delirium subtypes in hip fracture patients. The mean patient age was high (>84.8 years). HD prevalence ranged from 11.8% to 41%. Interestingly in the study by Lundström et al. [33] nondementia patients displayed significantly higher HD rates (41 versus 22%). In this study delirium subtypes were not a predictor of mortality during hospitalization and at 1 year. 

*Intensive Care Unit (ICU)*. The majority of the recently published studies (9 studies, Table 4) involve critically ill patients. All but one have used CAM-ICU and RASS for delirium detection and subtyping, respectively. HD prevalence rates were broadly ranged (36–100%) and the development of HD was associated with adverse short-term outcome in most studies [38–43]. Neurologic conditions were associated with HD in two studies [39, 44]. 

*Internal Medicine Wards*. Three studies presented in Table 5 assessed delirium in medical wards, involving elderly patients. All used the CAM for delirium diagnosis but different subtyping method and each study had a different primary objective. The larger study [46] was conducted in India and reported the highest HD prevalence (65%) in patients with severe medical conditions. 

*Palliative Care Setting*. Delirium in cancer patients was investigated in six recent studies (Table 6). Several diagnostic and subtyping methods were used by researchers. Prevalence ranged broadly (20–53%) and HD had been associated with old age [49], benzodiazepine dose and poor prognosis [50], and recognition failure [53] in different studies. In one study delirium recall was not significantly different across subtypes [48]. 

*Other Settings and Mixed Patient Populations*. Seven studies involved mixed patients' population (Table 7). In most studies patients were very old. HD prevalence rates varied significantly (6% in general C-L psychiatry, 47.1% in long-term facilities, 83% in several hospital wards, and 92% in the emergency department). All studies used CAM or DRS-R-98 for delirium diagnosis, but different subtyping methods.

Table 8 summarizes the results of HD prevalence across various wards. As shown in Table 8 and in Tables 1–7, there are significant differences among studies in methodology and patient populations, probably accounting for prevalence differences between settings. We preferred to include all recently published studies in order to present data from a range of clinical settings and to point out such methodology inconsistencies. If data were pooled from studies that were using the same diagnostic and subtyping tools and were performed in the same clinical settings, the number of studies included in this review would be small. Moreover, studies would mostly involve the ICU setting, in which methodology was consistent and the review would be less informative.

## 4. Discussion

This review comprised studies published during recent 6 years and shows that there is a growing literature on delirium subtypes. This may indicate the increasing awareness and interest of clinicians on this neuropsychiatric condition and the forms it may show.

### 4.1. HD Prevalence in Different Treatment Settings

Although HD prevalence varies considerably among settings (Table 8), it seems to be more common in the ICU and in cardiosurgery units where patients are very ill. High HD prevalence was also reported by a small study involving elderly patients who had been examined in an emergency department. In this report most (78.3%) HD cases were not recognized by emergency physicians [55]. Bearing in mind that delay in delirium recognition and treatment may increase morbidity and mortality it is conceivable that patients in these settings should be regularly assessed for signs of hypoactive delirium. HD prevalence is lower in adult patients examined by C-L psychiatric services and in mixed patient populations. Taken together these observations may suggest that the hypoactive variant of delirium may be associated with the severity of the underlying disorder and the patient's condition. In one of the original studies on delirium subtypes Liptzin and Levkoff [61] suggested that the hyperactive subtype may consist of patients who are physically well enough to get agitated, thus implying that hypoactive delirium may be an indicator of the severity of the patients' medical condition. This is also in agreement with the suggestion that older age may be a predisposing factor for the development of HD [62] and may indirectly support the view of this subtype as a severity indicator. Elderly patients are likely to suffer from several medical conditions and may have less physical strength than younger patients. For instance, the large proportion of hypoactive delirium found in the study on long-term facilities' patients [58] could be partly attributed to the very advanced mean age (almost 90 years) of participants. However, in two C-L psychiatry studies involving elderly patients, HD prevalence was found to be lower (6.25 and 14%, resp.) [28, 29] compared to the prevalence reported by other studies involving younger patients (19.9 and 30%, resp.) [30, 31]. These four studies used inconsistent methodology, and this may probably account for the observed differences.

### 4.2. The Prognostic Significance of HD

Few recent studies have addressed the prognostic significance of HD. In one study [24] HD was found to be an independent predictor of prolonged mechanical ventilation and ICU stay in cardiosurgery patients. In critically ill patients several studies reported higher short- [39–41] and longer-term [38] mortality of HD patients. HD was also associated with worse functional outcomes and worse quality of life in the long-term [42]. In cancer patients HD was associated with shorter survival by 1 month [50]. One study on hip fracture patients yielded no differences in mortality between delirium subtypes during hospitalization and at 1-year follow-up [33]. In patients at long-term facilities no differences in survival among delirium subtypes were shown [58]. In a postacute care study mortality was found to be increased in patients suffering severe delirium in the nondementia patient group, regardless of the subtype. In the dementia patient group mortality was higher in hypoactive-severe delirious patients [54].

Although data are scarce it could be argued that recent research is in line with previous published evidence suggesting that HD is associated with adverse prognosis. Hypoactive delirious patients have been previously reported to have higher mortality and disability rates and poor functional outcome. Current evidence suggests that HD may be associated with adverse outcomes in severely ill patients, but not in less severely ill patients. However it is not clear what the exact association of HD with poor outcome is. It may be that the outcome is associated with the underlying medical condition, which may be more severe in hypoactive delirious patients, or the delay in recognition and management and the complications of this subtype such as pressure sores and nosocomial infections [63] may explain better the poor outcome. Or it may be that cases of hyperactive delirium are readily recognized because symptoms such as agitation and disruptive behavior attract physicians' attention and lead to prompt treatment.

### 4.3. Other Findings of Studies in HD Patients

In one report [24] higher hemoglobin before surgery was associated with a lower prevalence of HD in cardiac surgery patients. Interestingly, in the same study a history of depression was a predictor of HD development. Two studies in which HD prevalence was high (>90%) found an association of HD with benzodiazepine use [26, 27] as well as a study in the palliative care setting [50]. Such an association was not found by others [29]. Two studies in C-L psychiatry services yielded contradictory findings regarding patients' cognitive symptoms [30, 31]. In a study in hip fracture elderly patients no differences in cognitive impairment between different subtype delirious patients were observed [32]. A larger study by Grover et al. [31] reported perceptual disturbances, delusions, and lability of affect to be significantly less common in HD patients. In another report in palliative care no differences in symptomatology between delirium subtypes were observed [52]. Some ICU studies reported an association of HD with serum max CRP levels [37], anemia [38], and neurologic disorders [39, 44]. Cerebrovascular disorders were associated with HD development in a large study in internal medicine wards in which a high prevalence (65%) of HD was recorded [46].

In general data are inconclusive, probably due to inconsistencies in studies' design and objectives and due to differences in research questions. However, the association of HD with sedative agents use, such as benzodiazepines, is a reproducible finding in several studies. This is clinically relevant and should be inquired for by clinicians during the diagnostic work-up in possible HD cases. Benzodiazepine use should be spared only to a minority of patients during hospital stay.

### 4.4. Methodological Considerations

It should be noted that there is a significant heterogeneity among studies in terms of scales used and participants' inclusion criteria. In several studies the assessment of delirium subtypes was made among other primary objectives, such as the test of an instrument's validity, or the estimation of physicians' or nurses' awareness of delirium. It appears that several preexisting limitations in research, such as differences in populations studied and inconsistencies in diagnostic and subtyping methods, pointed out by Meagher [13], have not been adequately addressed by recently published studies. However, it is unclear whether differences in HD prevalence within settings may be attributed solely to these inconsistencies. All instruments that have been used are reliable and valid and have been derived from Diagnostic and Statistical Manual of Mental Disorders (DSM) diagnostic criteria. Furthermore, subtyping instruments correspond to Lipowski's original description. Perhaps differences in studies' design and inclusion/exclusion criteria are more relevant and may better explain the observed differences. In several studies a prior diagnosis dementia or cognitive impairment was an exclusion criterion. In one report dementia patients were less likely to develop HD [33]. Conceivably such exclusion may bias the results toward an overrepresentation of HD cases. However, in another report from a geriatric monitoring unit rates of dementia were not different among patients with different delirium subtypes [59]. Future research should not exclude patients with dementia and cognitive impairment in order to clarify this issue by studying more representative samples of patients.

### 4.5. Future Research Directions

Data regarding treatment interventions for HD are scarce. Although several studies have examined the use of antipsychotics for the management of delirium symptomatology [64, 65], research on HD symptom management is limited. In a recent report it was found that symptoms of hypoactive delirious patients responded better than symptoms of hyperactive patients to the atypical antipsychotic aripiprazole [66]. Other research [51] suggested that hypoactive and mixed subtypes differed with regard to administrated chlorpromazine-equivalent doses, in line with a previous report which found that administrated haloperidol equivalent daily doses were higher in hyperactive delirium patients comparing to those with HD [67]. Interestingly all these studies were conducted in palliative care settings. More research in all treatment settings is needed and future studies should use consistent definitions and uniform scales and other measures to ensure comparability. Notably, according to Inouye et al., present evidence does not support the use of antipsychotics for prevention or treatment of delirium, and treatment should be reserved only for the small proportion of patients with severe agitation and distress, who pose a substantial risk of harm or interruption of essential medical therapy [68].

Another field for future research could be the primary care setting. It is noteworthy that despite the ongoing research on delirium subtypes there are no prospective studies on this issue in the primary care setting. This may be because primary care patients are less severely ill and delirium is generally infrequent in nonacute care settings. Few previous studies have investigated delirium in community dwelling patients, and it seems that delirium is uncommon in this population, including the nondemented elderly [69]. There is only one primary care study on delirium subtypes [70]. In this report delirium cases were examined retrospectively (1.1% rate in the clinical population) and delirium subtypes were determined with the application of Liptzin and Levkoff criteria. Of 9 patients diagnosed with delirium, only one hypoactive case could be determined (11%). Although this study has several limitations and was not included in this review, the results are in accordance with the suggestion that the physically stronger delirious patients, such as outpatients, are less likely to present the hypoactive type. Clearly more research is needed in primary care patient populations. It is unknown whether primary care physicians are familiar with the recognition of HD but research on common mental disorders suggests that these may often go undetected [71] in primary care settings, and this is probably the case of HD as well.

## 5. Conclusions

There is a growing recent research on delirium subtypes but evidence is still inconclusive with regard to symptom presentation and associations of the hypoactive variant. More research is needed and should expand to the primary care setting, where evidence is lacking. Treatment interventions for HD, including the use of atypical antipsychotics, should also be studied. Future studies should use more consistent methodology so that results can be comparable and reproducible.

Based on the findings of this review, it is proposed that all delirious patients should be regularly assessed for signs of HD in the everyday clinical practice. Today there are several valid and easily applicable instruments for the recognition of the hypoactive variant of delirium [72, 73]. The improvement of case identification would facilitate treatment and research.

## Figures and Tables

**Table 1:** HD in anesthesia and cardiac surgery.

Study	*N*, mean age in years	Diagnosis of delirium	Subtyping	Summary of findings
Ceriana et al., 2010 [23] (step-down unit)	18, 74.7	CAM-ICU, ICDSC	Criteria used by Panharipande et al.	61.1% HD

Stransky et al., 2011 [24] (cardiac surgery)	54, 71.1 mean age for HD patients	CAM -ICU, ICDSC	RASS	HD 77.8% Comparisons were made between HD patients and nondelirious subjects. Higher hemoglobin before surgery was associated with a lower prevalence of HD. HD was an independent predictor for prolonged mechanical ventilation time and ICU stay. A history of depression, duration of aortic clamping, the use of extracorporeal circulation, and preoperative medication with diuretics were predictors of HD after cardiac surgery whereas medication with b-blockers was associated with a significant lower prevalence of HD

Shaughnessy, 2013 [25] (cardiothoracic critical care unit)	23, age not available	CAM-ICU	RASS	73.9% HD

McPherson et al., 2013 [26] (cardiovascular ICU)	53, mean age not available	CAM-ICU	RASS	HD 91% In most cases the duration of delirium was less than 1 day Benzodiazepine use at admission was independently predictive of a 3-fold increased risk of an episode of delirium

Card et al., 2014 [27] (anesthesia)	124, 57	CAM-ICU	RASS	HD 56% at admission; 92% during stay in the PACU High opioid doses contributed to additional sedation, thus potentially contributing to HD features

CAM-ICU: Confusion Assessment Method for the Intensive Care Unit.

RASS: Richmond Agitation-Sedation Scale.

PACU: Postanesthesia Care Unit.

ICDSC: Intensive Care Delirium Screening Checklist.

HD: hypoactive delirium.

ICU: Intensive Care Unit.

**Table 2:** HD in consultation-liaison psychiatry.

Study	*N*, mean age in years	Diagnosis of delirium	Subtyping	Summary of findings
Singh et al., 2009 [28]	32, mean age not available, most cases >60 years	ICD-10	Liptzin and Levkoff criteria	6.25% HD

Sagawa et al., 2009 [29] (this study involved cancer patients that had been admitted in general medical wards)	100, 68	DRS-R-98	Liptzin and Levkoff criteria	14% HD Motor subtypes of delirium were not associated with any of the examined etiological factors (inflammation, dehydration and sodium abnormality, metabolism abnormality, benzodiazepine use, etc.)

Mushtaq et al., 2014 [30]	40, 27.8	MMSE, MDAS	No specific scale	30% HD; HD patients had more cognitive disturbances compared to hyperactive patients

Grover et al., 2014 [31]	321, 49	DRS-R-98	DMSS	19.9% HD Perceptual disturbances, delusions, and lability of affect were significantly less common in HD patients, compared to the hyperactive or mixed delirium group. There were no significant differences for the cognitive symptoms in DRS-R-98 across the different motor subtypes HD patients significantly less frequently received psychotropic medications, compared to patients with other delirium subtypes

ICD-10: International Classification of Disease.

MMSE: Minimental State Examination.

MDAS: Memorial Delirium Assessment Scale.

DMSS: Delirium Motor Subtype Scale.

DRS-R-98: Delirium Rating Scale-Revised-98.

HD: hypoactive delirium.

**Table 3:** HD in patients with hip fractures.

Study	*N*, mean age in years	Diagnosis of delirium	Subtyping	Summary of findings
van Munster et al., 2009 [32]	62, 84.8	CAM, DOS, DRS-R-98	DSI	Delirium subtype was specified in 34 delirious patients. 11.8% HD No significant difference in S100B protein or Neuron Specific Enolase levels was seen between delirium subtypes. There were no differences in cognitive impairment among different subtype delirious patients

Lundström et al., 2012 [33]	129, 86	MMSE, OBS scale	OBS scale	22% HD in dementia patients 41% HD in nondementia patients No differences in mortality between delirium subtypes during hospitalization and at one-year follow-up

Slor et al., 2013 [34]	42, 87.6 for HD patients	CAM, MMSE	DRS-R-98	16.7% predominantly HD. 36.7% had a variable profile. Delirium subtype at these assessments was hypoactive in 25% of cases (28 out of 112 assessments)

Slor et al., 2014 [35]	46, 86.3	CAM, MMSΕ	DMSS, DRS-R-98	23.9% HD

CAM: Confusion Assessment Method.

OBS scale: Organic Brain Syndrome scale.

DMSS: Delirium Motor Subtype Scale.

DSI: Delirium Symptom Interview.

DOS: Delirium Observation Screening.

MMSE: Minimental State Examination.

DRS-R-98: Delirium Rating Scale-Revised-98.

HD: hypoactive delirium.

**Table 4:** HD in the Intensive Care Unit.

Study	*N*, mean age in years	Diagnosis of delirium	Subtyping	Summary of findings
Guenther et al., 2010 [36]	25, 74	CAM-ICU	RASS	80% HD

Tsuruta et al., 2010 [37]	21, 70	CAM-ICU, ICDSC	RASS	100% HD in nonventilated patients. Serum max CRP levels and length of ICU stay were independent associations of delirium development

Robinson et al., 2011 [38]	74, 64	CAM-ICU	RASS	68% HD; only 1% hyperactive Subjects with HD were found to be older and more anemic and had higher six-month mortality in comparison to subjects with mixed type delirium. Sacral skin breakdown occurred more frequently in HD patients than in the mixed delirium subjects

van den Boogaard et al., 2012 [39]	411, 64	CAM- ICU	RASS	36% HD HD prevalence was significantly higher in the neurology and neurosurgery group. Significantly more patients with a hypoactive and mixed subtype died compared to the hyperactive subtype

van den Boogaard et al., 2012 [40]	171, 65	CAM-ICU	RASS	36.8% HD Survival was significantly lower in HD and mixed delirium patients compared to hyperactive delirium subjects At 18 months after discharge HD patients performed significantly better on the domain mental health than mixed or hyperactive patients

Sharma et al., 2012 [41]	54, 49.5	DRS-R-98	RASS	45.3% HD HD was a predictor of mortality in patients with delirium

Naidech et al., 2013 [42]	31, 63	CAM-ICU	RASS	90% HD Delirium symptoms were brief (1 day) in duration and were associated with longer length of stay, subsequent worse functional outcomes, and domain-specific QOL, compared to nondelirious subjects

Caruso et al., 2014 [43]	163, 59	CAM-ICU	RASS	66.3% HD Delirium subtype was not associated with ICU bed design (multibed or single-bed)

Leite et al., 2014 [44]	34, 40.8	CAM-ICU	RASS	73.5% HD Youngest patients with neurologic trauma who were in the process of being weaned from mechanical ventilation were more inclined to present with HD

CAM-ICU: Confusion Assessment Method for the Intensive Care Unit.

RASS score: Richmond Agitation Sedation Scale score.

DRS-R-98: Delirium Rating Scale-Revised-98.

ICDSC: Intensive Care Delirium Screening Checklist.

ICU: Intensive Care Unit.

HD: hypoactive delirium.

**Table 5:** HD in internal medicine.

Study	*N*, mean age in years	Diagnosis of delirium	Subtyping	Summary of findings
van Munster et al., 2010 [45]	126, 81.6	CAM	DSI	Delirium subtype was specified in 38 patients. 18.4% HD There were no differences in S100 levels in patients with different subtypes of delirium

Khurana et al., 2011 [46]	400, 70.8	CAM Hindi MMSE	Criteria proposed by Lipowski	65% HD Cerebrovascular diseases, congestive heart failure, malaria, liver failure, and electrolyte imbalance were associated with HD development

Franco et al., 2014 [47]	34, 78.3	CAM, MMSE	DRS-R-98	38.2% HD There was an association of impairment in temporal orientation, spatial orientation, and visuoconstructional ability at admission (estimated with the MMSE) with the development of HD and mixed type

CAM: Confusion Assessment Method.

Hindi MMSE: a vernacular (Hindi) version of the Minimental State Examination.

DRS-R-98: Delirium Rating Scale-Revised-98.

MMSE: Minimental State Examination.

DSI: Delirium Symptom Interview.

HD: hypoactive delirium.

**Table 6:** HD in palliative care.

Study	*N*, mean age in years	Diagnosis of delirium	Subtyping	Summary of findings
Bruera et al., 2009 [48]	99, 60	MDAS, MMSE	No specific scale	20% HD Delirium recall was not significantly different according to subtype (hyperactive versus hypoactive versus mixed)

Godfrey et al., 2010 [49]	25, 76.5 (HD patients)	DRS-R-98, CTD	New subtyping scheme derived from Meagher	40% HD HD patients were older than those in each of the other groups

Meagher et al., 2011 [50]	100, 70.2	DRS-R-98	DMSS	28% HD HD was associated with a poorer prognosis, regarding survival at 1 month, compared to other subtypes Transitions into hypoactive subtype were preceded by increased benzodiazepine dose

Leonard et al., 2011 [51]	100, 70.3	CAM, DRS-R-98, CTD	DMC	33% HD Hypoactive and mixed subtypes differed with regard to administrated chlorpromazine-equivalent doses (hypoactive patients received lower doses)

Boettger and Breitbart, 2011 [52]	100, 58.3	MDAS	MDAS	53% HD There were no differences in symptomatology between subtypes

Rainsford et al., 2014 [53]	22, 70.1	CAM, DRS-R-98	DRS-R-98	50% HD The treating team failed to recognize 45.5% of those with HD

CAM: Confusion Assessment Method.

DMSS: Delirium Motor Subtype Scale.

DRS-R-98: Delirium Rating Scale-Revised-98.

MDAS: Memorial Delirium Assessment Scale.

MMSE: Minimental State Examination.

CTD: Cognitive Test for Delirium.

DMC: Delirium Motoric Checklist.

HD: hypoactive delirium.

**Table 7:** HD in other wards.

Study	*N*, mean age in years	Diagnosis of delirium	Subtyping	Summary of findings
Yang et al. 2009 [54] (postacute care)	441, 84.1	CAM, MDAS, MMSE	DSI	46.7 HD Increased mortality was found in the hypoactive-severe and hyperactive-severe classes in the nondementia patient group and also in the hypoactive-severe class in the dementia group

Han et al., 2009 [55] (emergency department)	25, 80 (median age)	CAM-ICU	RASS	92% HD Most (78.3%) HD cases were not recognized by emergency physicians

Scheffer et al., 2011 [56] (hip fracture and medical patients)	41 patients with hip fracture (mean age 86.7) 56 medical patients (mean age 82.1)	CAM DOS scale, DRS-R-98	DSI	14.3% HD (data from 56 patients)

Rice et al., 2011 [57] (several hospital wards)	12, 80.1	CAM, MMSE	Liptzin and Levkoff criteria	83% HD. Most HD patients were admitted to the hip-fracture service

DeCrane et al., 2011 [58] (long-term facilities)	70, 89.9	CAM, MMSE, CAC-A, NEECHAM confusion scale	CAC-A	47.1% HD Pulmonary disorders were the leading underlying cause of death in the hypoactive subgroup No differences in survival among subtypes

Chong et al., 2013 [59] (geriatric monitoring unit)	228, 84.2	CAM, DRS, MMSE	Not specified	18.4% HD Rates of dementia were not different among delirium subtypes. Patients with HD and mixed type had more comorbidities than hyperactive patients There were benefits of bright light therapy as part of a multicomponent delirium management program for all patients

Meagher et al., 2014 [60] (palliative care, adult and old age C-L psychiatry services)	375 Mean age not available	DRS-R-98	DMSS	32% HD in palliative care 21% HD in old age C-L psychiatry 6% HD in general C-L psychiatry

CAC-A: Clinical Assessment of Confusion.

CAM: Confusion Assessment Method.

CAM-ICU: CAM for Intensive Care Unit.

RASS: Richmond Agitation-Sedation Scale.

DOS scale: Delirium Observation Screening scale.

DRS: Delirium Rating Scale.

DRS-R-98: Delirium Rating Scale-Revised-98.

DMSS: Delirium Motor Subtype Scale.

DSI: Delirium Symptom Interview.

MDAS: Memorial Delirium Assessment Scale.

MMSE: Minimental State Examination.

**Table 8:** HD prevalence in different settings.

Setting	Patients' mean age	HD prevalence
Anesthesia and cardiac surgery	57–74.7	56–92%
C-L psychiatry	27.8–68^*∗*^	6.25–30%
Hip fractures	84.8–87.6	11.8–41%^*∗∗*^
ICU	40.8–74	36–100%
Internal medicine	70.8–81.6	18.4–65%
Palliative care	58.3–76.5	20–53%
Other	80.1–89.9	6–92%^*∗∗∗*^

^*∗*^This mean age involves cancer patients.

^*∗∗*^This prevalence refers to nondementia patients.

^*∗∗∗*^This prevalence refers to emergency department patients.
